# Cyborg Children: A Systematic Literature Review on the Experience of Children Using Extended Reality

**DOI:** 10.3390/children11080984

**Published:** 2024-08-14

**Authors:** Marina Everri, Maxi Heitmayer

**Affiliations:** 1 Room C322—Health Sciences Centre, School of Medicine, University College Dublin, Belfield Campus, Dublin 4, D04 V1W8 Dublin, Ireland; 2 London College of Fashion, University of the Arts London, London WC1V 7EY, UK; m.heitmayer@fashion.arts.ac.uk; 3 London School of Economics and Political Science, London WC2A 2AE, UK

**Keywords:** virtual reality, augmented reality, children, extended reality (XR), Leximancer, systematic literature review

## Abstract

This literature review presents a comprehensive and systematic account of research on the experiences of children with extended reality (XR), including VR, AR, and other types of immersive technologies that enhance and augment children’s activities. The search on Scopus and Web of Science produced 531 outputs. Content analysis with inter-rater reliability (Krippendorff’s α) and Leximancer, a software for text mining, were used for analyzing the material. Four research strands were identified: (1) interventions, treatments, and medical procedures in clinical contexts; (2) teaching and learning enhanced by XR; (3) children’s adoption and user experiences; (4) design and prototyping of XR hardware and software for children. The results showed the following findings: (a) studies on children’s clinical interventions and treatments using HMD-supported immersive virtual reality comprise the most substantial strand of studies; (b) research in this area, and in teaching and learning studies, has grown dramatically since 2017, while the other areas have been stagnant over the years; (c) AR research is still limited and is mainly applied in educational contexts for design and prototyping; (d) few studies have considered children’s perspectives on XR safety issues; (e) research on the use of XR for enhancing social and emotional skills development is underrepresented. Future research should focus on the potential of XR technologies for interventions to enhance children’s psychosocial wellbeing and health more broadly. The further implications and study limitations for the fast-developing nature of this transdisciplinary research field are also discussed.

## 1. Introduction

The Cyborg—the augmentation of the human body through the integration of technological components—is a fictional creation epitomized in science fiction shows and novels (e.g., Star Trek and William Gibson’s and Philip Dick’s works) that is increasingly becoming a reality. The sight of people (including children) wearing technology monitoring their bodily functions (smart watches, fitness trackers), recording their environments (action cameras), or feeding inputs directly into sensory organs (headphones, smart glasses) has become familiar (and in many cases, more frequent than those who do subject their bodies to some form of technological augmentation). As envisioned by Donna Haraway [1], it seems that the boundaries between the human and the machine, the physical and the virtual, have progressively blurred. This has generated an embodiment of technologies in humans’ bodies and lives more broadly and has altered human’s sensory perceptions as well as the way in which they engage with their environments.

Alongside these physical and cognitive transformations, big tech companies have competed to bring cutting-edge technologies to the market: after an announcement from Meta in June 2023 that they were going to invest millions in the Metaverse, Apple released the Apple Vision Pro, a device which allows for the combination of physical, virtual, and augmented reality. These changes, however, do not come without controversies: the Metaverse, which is envisaged to allow us to move away from the Internet as a distinctly separate space from our physical world, and virtual reality (VR), which is one of the possible ways we will access the Metaverse, have reignited debates on privacy, safety, and surveillance, especially when children are concerned [2,3,4]. In fact, the rapid development and cost accessibility of immersive technologies as well as the fast scaling of VR products in the gaming industry have made children the main target for the commercialization of devices and programs for experiences in virtual environments [5,6].

Research on VR started more than forty decades ago and has evolved together with hardware and software development (for an overview see [7,8,9,10]); however, it is only in the last decade that studies started to investigate the opportunities and challenges of immersive technologies for children’s cognitive and social–emotional development, health, and wellbeing (e.g., [11,12,13]). There has been growing interest in the study and application of VR for children’s clinical treatments, enhancement of learning skills, and entertainment, as signaled by the recent literature reviews [14,15,16,17,18,19] and research reports (e.g., [13,20]) that are available in each of these areas. However, an initial review of this fast-growing transdisciplinary area of research (psychology, computer science, and education) signals inconsistencies on the use of the terms and concepts in VR research: VR can occasionally be confused with terms like “mixed reality” (MR), “augmented reality” (AR), and “augmented virtuality” (AV), which are used differently in research and practice [21]. The use of the term ‘Extended Reality’ (XR) was recently proposed to refer to a more open approach to different forms of immersive technologies that extend and modify the perception of physical environment, to acknowledge the ‘unknown’ variable [22]. In this sense, we concur with Raushnabel et al. [22] (p. 2) that ‘the extant literature is ripe for reorganizing and reconceptualizing’ the studies on VR and other similar technologies aimed at simulating, expanding, or modifying reality. Therefore, together with the need for terminological clarifications, we wanted to provide a ‘reorganization’ of research strands on VR involving children to identify research areas that require further development for a better understanding children’s experiences with immersive technologies.

Informed by a research framework focused on children’s rights in the digital environments [23] and by a revisited ecological theory that acknowledges the centrality of both physical contexts and virtual contexts in child development [24], the current literature review aimed at providing an accurate and comprehensive account of the development of the field researching children’s experiences with VR and related technologies. As suggested by recent studies [22], in this paper, we used the term XR to refer to the range of technologies that modify reality, i.e., the perceived physical environment (e.g., VR, AR, CAVE, etc.), which are available in the market and used in research with samples of children. More specifically, the goals that guided our analysis of the literature were as follows:

1. Identify specific research strands that have studied children’s experiences with XR technologies.
2. Examine the different types of XR technologies (hardware and software) and their applications across the different research strands.
3. For each strand, discuss new directions for future research with XR that involve children (under 18 years of age) as research participants or stakeholders.

## 2. Method

We chose the scoping review method as a systematic and explorative approach to search through and analyze the academic literature [25] and followed the general PRISMA guidelines for systematic literature reviews [26,27]. Given the differences in epistemological and methodological approaches adopted in the literature, as well as the great variety of operationalization and phenomena relating to children’s experiences with XR, neither a meta-analytic approach nor a confirmatory analysis of the material were appropriate [28,29,30,31]. Instead, a detailed content analysis of the findings and the coding and categorization of the pertinent literature is presented.

### 2.1. Keywords and Literature Search Terms

Based on an initial, unstructured search and a reading of the literature, we noted great diversity used in terminologies both across and within scientific disciplines. Given the scoping outlook of this review, we opted for a broad and inclusive set of search terms to try and capture as many different types of children’s experiences with XR as possible. Therefore, the key terms for the initial screening included references focused on children’s samples (under 18 years of age) and referred to immersive/augmented/virtual reality environments (see Table 1).

The literature search was conducted in July and August 2022 on Scopus and Web of Science, which cover a broad range of the literature and complement each other well [32,33]. This resulted in an initial set of 12,109 papers.

### 2.2. Screening and Coding Process

**Figure 1:**
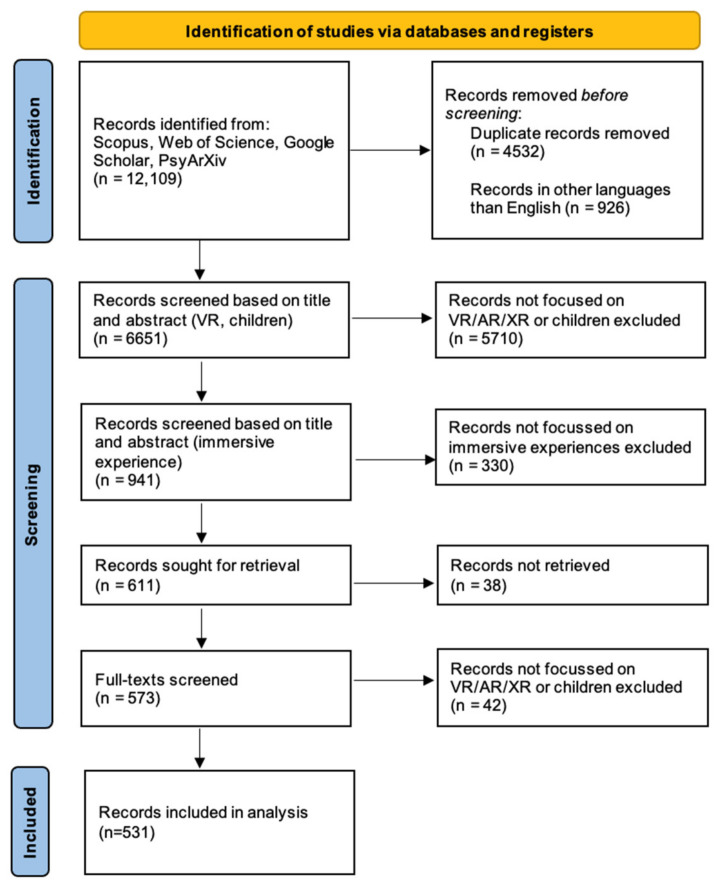
Prisma diagram on screening of the literature on children and XR.

The screening of the material was iterative rather than linear and was carried out following the steps presented in Figure 1 below. First, titles and abstracts were screened and duplications and records in any non-English language removed. Therefore, the material was reduced to a set of original 6651 unique papers. Second, papers not focused on children and VR/AR or mixed reality (MR) were excluded (*n* = 5710). Third, from the remaining *n* = 941 papers, records not focused on immersive experiences were excluded (*n* = 330). Fourth, 38 out of the remaining 611 records sought for retrieval could not be retrieved even through the library service and our institutions. Therefore, 573 full texts were screened in depth (full text) and 42 final records were excluded because of a lack of focus on immersive technologies or children. Hence, 531 records were fully reviewed and analyzed using content analysis. Two researchers worked independently throughout the screening process and had repeated meetings to discuss and agree on the selection criteria and the final set of records (see Supplementary Materials for the full list of papers).

### 2.3. Analysis of the Material

The analysis of the full-text records was conducted on a final corpus of 531 resources. We used content analysis to categorize and quantify the material surveyed to provide an overview of research strands on the topic of the review [34,35]. Once the records were categorized, we proceeded to examine the different types of children’s immersive experiences presented in the various papers. Data were double-coded independently by the authors and the codes were then developed jointly over three interactions.

Following an initial review, we identified four groups of papers: *Clinical Interventions*, *Teaching and Learning*, *Adoption and User Experience*, and *Design and Prototyping* (and one ‘other’ category, including relevant literature reviews). In a second independent round of coding the papers, we find an inter-rater agreement of 96.7% using Krippendorff’s α. The remaining disagreements were resolved in discussion in the final and third round.

Given the extensive number of resources and in order to provide further validity to our content analysis, we used Leximancer, a software that uses an unsupervised machine learning algorithm for text mining to identify concepts, their frequencies, and their co-occurrences with other concepts.

## 3. Findings

### 3.1. Strands of Studies on Children and XR

**Figure 2:**
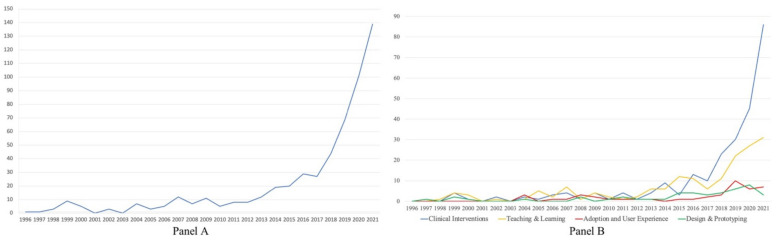
Research interest in the topic from 1996 to 2021. (**Panel A**) presents combined figures and (**Panel B**) presents figures across the four research strands.

An analysis of the year of publication of the identified resources provided evidence on the rapid increase in studies over the last decade: since 2012, the number of studies has gradually grown, with a significant curve starting from 2017 (Figure 2, Panel A).

**Figure 3:**
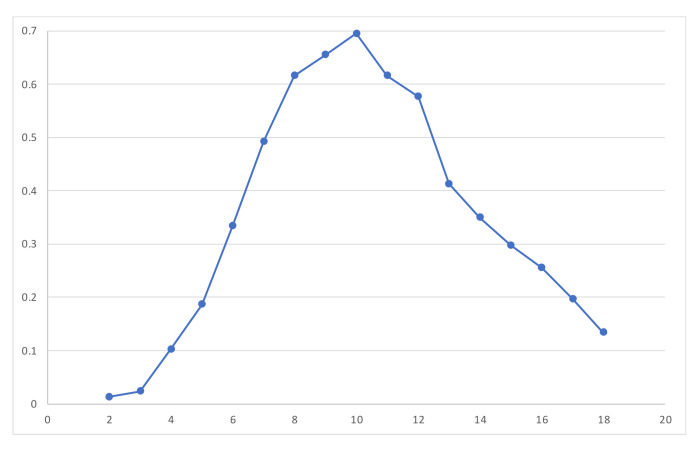
Percentage of studies including children in their sample by age group.

Studies included immersive experiences for children across various stages of development ranging from 2 to 18 years old. Roughly, 71.6% of studies carried out primary empirical work with children; the rest of the studies worked theoretically or reviewed the literature. We observed a wide spread of age ranges that were included in studies, suggesting that the development and use of XR applications are considered appropriate for children at all ages. Figure 3 gives an overview of the percentages of studies including each age group in the sample by children’s age; 58–70% of all studies included children aged 8–12, respectively. This age group thus emerged as the most studied population when it comes to children’s experiences with XR. It is important to note here that some studies extended the definition of children slightly and included young adults over the age of 18, often those who had developmental disorders or disabilities. Given the focus of our review, we have not included those numbers in the overview of the results.

The analysis of the collected material allowed us to identify four categories of studies, or research strands, which involved children (under 18 years of age) as participants or stakeholders in the study: (1) interventions, treatments, and medical procedures in clinical contexts; (2) teaching and learning enhanced by immersive technologies; (3) children’s adoption (UX) and experiences with immersive technologies; (4) design and prototyping of VR/AR/MR hardware and software for children. Interestingly, clinical interventions studies have dramatically increased from 2017 (Figure 2, Panel B); they are followed by teaching and learning studies, which have grown but to a much lesser extent; adoption and user studies, together with design and prototyping, have been stagnant over the years.

Twenty-nine resources across the four research strands were literature reviews, the majority of which (*n* = 24) pertained to the use of VR for medical procedures, treatments, and interventions (category 1), and 17 were short papers published by R&D teams (e.g., educational start-ups) that tested the development of software and hardware products for commercialization (category 4). The remaining resources (*n* = 485) were peer-reviewed, i.e., empirical research articles on XR and children. The distribution of these records across the four strands is reported in Table 2. For each research strand, we also indicated the type of immersive technology investigated.

More than half of the surveyed papers (*n* = 251) concerned research on XR for interventions, medical procedures, and treatments in clinical settings. One third of the studies pertained to the teaching and learning of different skills in educational contexts using XR (*n* = 153). Studies on children’s user experiences were *n* = 44, and the development and testing of prototypes was reported in *n* = 37 papers.

**Figure 4:**
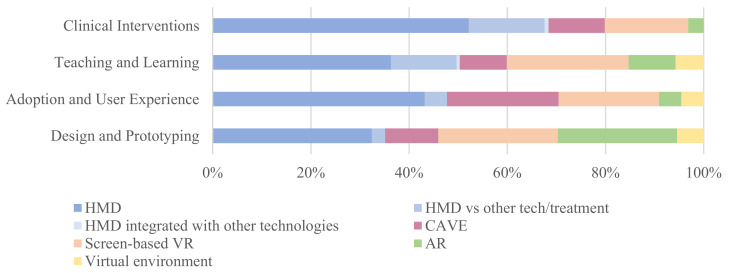
Overview of research strands and quantification of studies based on type of technology considered in the studies.

When considering the type of hardware examined in the different articles (Figure 4), HMDs emerged as the most frequently studied hardware across the different strands, with most studies pertaining clinical contexts (*n* = 131). Similarly, CAVE provides fully immersive experiences; on this technology, there were 29 articles for the clinical strand, 15 articles for the educational strand, 10 articles for design and prototyping, and 4 articles for the adoption/user experience strand. Other research focused on VR supported by 2D screens: a slightly higher number of research studies were carried out in clinical settings with clinical samples (*n* = 43) compared with research in educational contexts (*n* = 39). A small number of studies focused on the use of AR, and, interestingly, they were mainly concerned with teaching and learning processes (*n* = 15) and design and prototyping (*n* = 9).

### 3.2. Specific Topics and Concepts in Research on Children and XR

The Leximancer analysis resulted in 47 unique concepts and 12 distinctive themes (note that themes are not mutually exclusive, and all papers related to multiple themes): body, children, crossing, design, environment, intervention, learning, parents, patients, risk, social, and VR. Unsurprisingly, the most dominant themes were as follows: VR, with 31,870 occurrences across the literature; children, with 25,574 occurrences across the literature; environment, with 17,807 occurrences across the literature.

**Figure 5:**
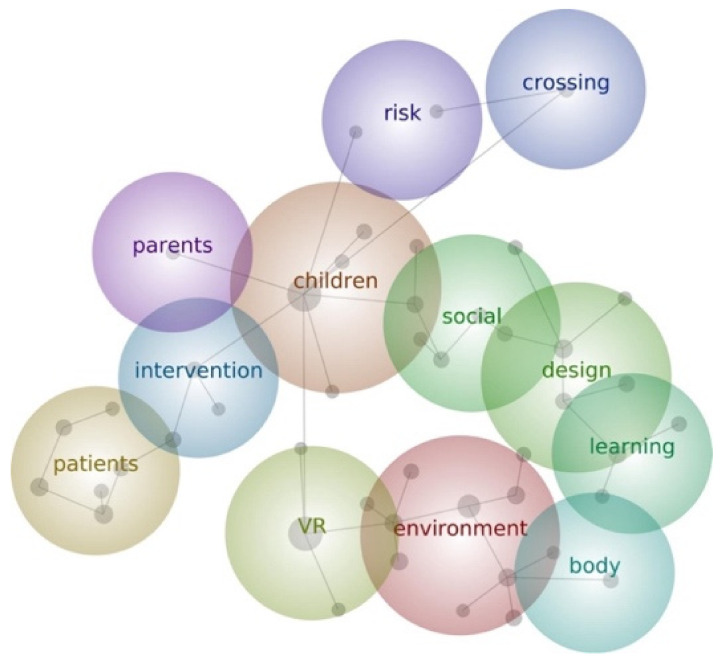
Thematic map resulting from the analysis with Leximancer.

Figure 5 shows the thematic map spatially outlining the relationship between themes. As an example, the theme of environment is closely linked with VR and body; body connects with learning; learning connects with design and social. This indicates a strand of studies that considered aspects related to virtual environments, children’s bodily experiences, learning, and social skills. Differently, themes less closely connected with VR and children’s immersive and bodily experiences are found in research on risks, such as street crossing.

Thematic visual maps present similarities with the four categories we have identified in the content analysis: the 12 distinctive themes can in fact be merged into content categories pertaining to (a) clinical interventions (intervention, patients, risk); (2) education and teaching and learning processes (crossing, learning, parents, social); (3) children’s experiences (body, children, environment); and (4) design and prototyping (design, VR). Therefore, Leximancer’s automated themes extraction approach supports the validity of the content analysis (QCA) (Table 3).

**Figure 6:**
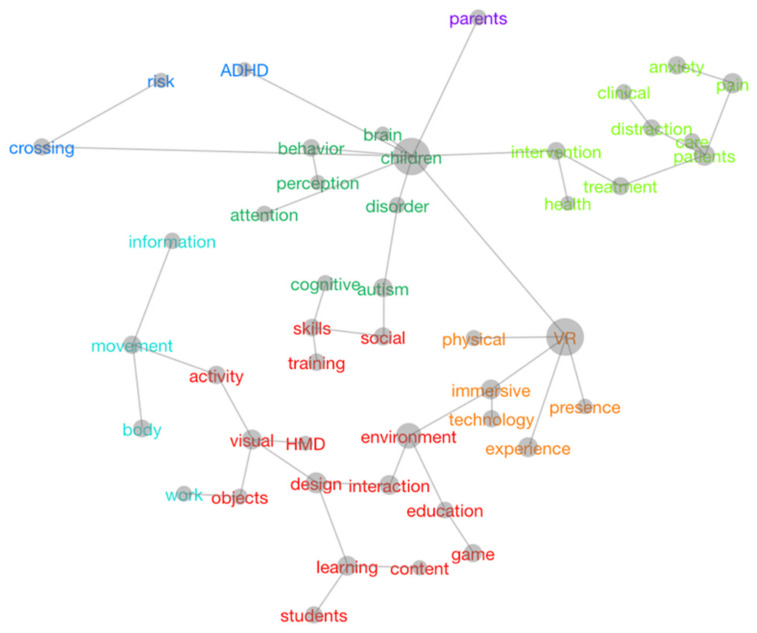
Conceptual map resulting from the analysis with Leximancer. Related concepts are highlighted in the same color.

Another important advantage of using Leximancer for literature reviews is the possibility of rapidly acquiring information on the relationships between concepts that define research themes. This provides more clarity about the content of the resources pertaining to a particular topic (Figure 6).

Continuing with the example of the theme of ‘environment’ and the connection with the other themes which fall in the broader category of teaching and learning (in red), we can see the detail of the topics investigated across different papers: school, educational games, learning/training, and skills. Several resources focused on using immersive technologies to enhance training for learning different school subjects, such as mathematics, geometry, foreign languages, music, etc. [36,37,38,39,40], and for the development of psychosocial skills, such as problem solving [41], safety, and protection, e.g., from fire [42,43] and road traffic, with substantial research carried out on road crossing simulations [44,45,46,47,48]. One paper only addressed Internet risks such as cyberbullying [49]. Other studies considered learning processes in terms of collaborative learning in the virtual space [50,51], experiential learning [52,53], and students’ motivation and engagement [54].

As for concepts pertaining to the most-represented category of studies, namely children’s clinical interventions, the Leximancer analysis provided more details on the types of treatments and the types of problems treated using XR, such as anxiety [55,56,57] and physical pain [58,59,60,61,62,63]; additionally, there was research on the use of XR for distraction in preparation for medical procedures, such as dentist surgeries [64,65], injections [66,67,68,69], or other painful medical procedures, especially in children’s oncology [70,71,72]. Additionally, and unsurprisingly, some of these papers focused on the treatment of perception and attention deficits linked to pathologies such as Autism [73,74,75,76,77] and ADHD [78,79,80]. Immersive technologies proved to be particularly effective for the treatments of these clinical issues. Interestingly, a recent article addressed the use of IVE for ADHD problems and risk behaviors, such as street crossing (in blue, Figure 6) [81]. Lastly, a cluster of studies part of this stand focused on rehabilitation procedures for cerebral palsy and similar neurological problems [82,83,84].

Information, movement, body, and work are topics found in research papers concerned with the category of design and prototyping (light blue, Figure 6). These papers are mainly scientific reports, conference proceedings, and short papers which illustrated the development of immersive technologies software and hardware for children. The content of these resources ranges from innovative wearable sensors for different parts of the body to track children’s movements or cognitive functions [85,86,87] to different types of XR software for educational (e.g., [88,89,90,91]) and entertainment purposes [92,93,94]. A substantial cluster of studies in this category also concerned the development of IVE serious games for enhancing children’s safety and protection [95,96,97,98,99,100] and physical skills, i.e., exergames [101,102,103,104]. One study focused on developing social games comparing samples of children and adults [105].

The remaining concepts of immersion, presence, and experience correspond to the category of adoption and user experience. Since the focus was on user’s perspectives, these papers examined key features of XR such as the sense of presence [106,107] and embodiment [108,109,110] that children felt while experiencing a virtual environment. Also, attention was given to children’s physiological (e.g., sickness and nausea, posture) and psychological (perception of distances and heights; sound, light, and colors; social cues, comfort, and satisfaction, etc.) experiences of being fully immersed in a virtual space that the brain treats as ‘real’ [111,112,113,114,115,116,117]. Two articles specifically investigated children’s experience of safety with VR technology [77,118]. Attention was also given to the impact of the virtual experience on physical space [119,120].

Lastly, Leximancer allowed us to identify a concept named ‘parents’ (in purple, Figure 6), linked with children’s experiences with XR. A closer analysis of the full text showed that there is no prevalence of these studies in one strand; instead, we found few resources distributed across the four categories: clinical [121], user experience [122,123], prototyping [124], and education [125]. These studies included samples of children and parents.

## 4. Discussion

This systematic literature review has provided an accurate account of research material concerned with XR and children under 18 years of age as study participants. Three research goals guided the analysis of the material: (a) identify specific research strands on XR and children; (b) examine which XR technologies were most represented in each strand and how XR approaches were applied; (c) discuss new directions future research within each strand. Results are discussed focusing first on the research interest on the topic and sampling characteristics and methods, and then on the discussion of the four identified strands, highlighting XR applications, gaps, and future directions for research.

The timeline of the publications is the first result that is worthy of notice: the analysis of the literature showed a rapid growth of studies on XR with samples of children between 2016 and 2017. This result could be attributed to the release of the first Oculus Rift, and to other HMDs becoming available in the consumer market at more affordable prices around that time. Consistently, the possibility of having access to affordable equipment together with the portability of HMD has prompted researchers to explore the applications of VR in different contexts such as hospitals, psychotherapy settings, homes, and schools, thereby enabling easier access to children as subjects for research in their everyday life environments.

The analysis of methods and sampling across the corpus of the surveyed papers allowed us to show that most of the published works were empirical studies, with a minority of conceptual papers and literature reviews. This suggests that researchers have included XR in their research studies with children to test hypotheses, evaluate XR effectiveness, and explore research topics concerned with XR in a range of child development domains (learning processes, psychopathology, informal learning and entertainment, etc.). Additionally, when considering the age range of participants, we observed that childhood and early adolescence are the most studied developmental periods. This confirms that XR can be useful to support different cognitive, social, and emotional functions during childhood. This is also the age in which play is core to child development, and VR in particular allows children to have fantastic immersive experiences and engage with different tasks (e.g., [49,54]). Future research could consider the application of this technology with younger children (under 8 years of age) or with older adolescents (14–18 years of age). The use of immersive technologies for gaming is well-established among adolescents (e.g., [6]); however, XR could be considered to enhance social–emotional skills and self-efficacy [126] in samples of older adolescents when peer groups processes become core to their identity development and wellbeing.

### 4.1. Clinical Interventions and HMDs

Our results showed a dominant category of studies on the use of HMDs for immersive virtual reality experiences used in clinical interventions with children. These studies ranged from children’s perception of pain reduction to children’s immersion in simulators for treatments of psychological [78,127] and neurological problems, e.g., cerebral palsy [82,84]. The advantages of using HMD for VR use in clinical settings have been demonstrated with samples of adults also [128,129,130]. Therefore, the application of immersive VR in clinical settings seems to emerge as a consolidated area of studies that applies to different samples across the lifespan. These results suggest that future research with children should continue to use immersive technologies for treating different types of physical and behavioral problems. Pediatricians, in particular, can consider the use of VR to enhance the effectiveness of their interventions; for instance, for distraction during painful procedures and examinations or for modifying children’s habits such as eating or exercising, especially for children with diabetes or weight issues. In other words, the affordability of HMDs and the availability of educational games and health-related contents make immersive technology a useful tool which can complement different types of medical interventions.

Our results also showed an overrepresentation of studies on XR for the treatment of ASD and ADHD; meanwhile, other social–emotional problems, such as children’s depression and anxiety, seem to be still peripheral. A possible explanation of a paucity of studies on XR and children’s social–emotional problems could be linked to the lack of knowledge on the possible ‘side effects’ and the ethical implications of XR treatments. In fact, if in adults’ samples anxiety and other psychological disorders have been effectively treated using VR (e.g., [131,132]), research involving children might be limited because of the amplifications of the emotional reactions triggered by VR treatments. This would require careful consideration of the content to which children are exposed as well as the ethical implications of the intervention protocols [20,133].

### 4.2. Teaching and Learning and AR

AR research is underrepresented compared with work that uses HMDs, and it is mainly represented in the category of education, specifically in studies focused on teaching and learning, and design and prototyping. As this technology often relies on apps that can be installed on tablets and smartphones, it is easily accessible for conducting research in various environments including schools. The strand of research in education seems a promising and expanding area of research for harnessing the potential of immersion for enhancing learning skills, in particular self-efficacy and students’ motivation and engagement (e.g., [54]). Interestingly, we found several studies that addressed the use of different types of XR for teaching road safety skills [44,45,46,47,48]. While this is a relevant safety issue for children, very few studies considered other import risks, such as violence and aggression, for which XR could be productively used to support the learning and further development of appropriate social and emotional skills [49]. We suggest that researchers and educators consider investigating these subjects further, especially when adolescents are concerned: gender issues, discrimination, aggression, and violence continue to be important challenges for youth in contemporary society. Programs that harness XR characteristics to complement children’s skills’ development can motivate children and make them more engaged with the content of their learning experience.

### 4.3. Adoption and User Experience

Despite user research (UX) becoming an integral part of technological development, few papers seem to reach the scientific community through a peer-review process (*n* = 44 compared with clinical interventions, *n* = 251, and teaching and learning, *n* = 153). For this strand of studies, as well as for design and prototyping (see next paragraph), we found a prevalence of conference papers. Few resources considered children’s adoption and use of XR technologies; these studies focused on physiological and psychological reactions related to immersion, sense of presence and embodiment, e.g., sickness and perceptions of distances, lights, etc. [106,110,111,117], and, to a lesser extent, children’s safety issues around the use of technologies [18,77]. The hardware per se does not seem to be problematic for children (whereas it appears to be more problematic for adults in terms of experiencing motion-sickness); it is rather the content and how technologies are used that can cause concern in terms of risks and safety. Interestingly, we could only find two studies that explored risks from a UX perspective [77,118] and no resources that explored children’s positive and negative experiences through in-depth qualitative and observational investigations of practices, opinions, and concerns.

### 4.4. Design and Prototyping: The Challenge of Commercialization

Studies on XR design and prototyping represent the minority of the surveyed studies (*n* = 37). There is no prevalence on the type of technology investigated. Together with studies on UX, they have not expanded over ten years (Figure 2, Panel B) and seem to be confined to a niche of research which is carried out alongside ‘traditional’ academic work. One potential explanation for the observed trend, namely the lack of resources despite the rapid growth of the sector and the technological developments, is that this research is concerned with commercially sensitive information, and that software and hardware development is in most cases financed by private companies. In this sense, children’s experiences remain in the realm of commercial sensitivity that is protected by patents, thereby leaving an important gap in the advancement of knowledge in this area. This is unfortunate as children, teachers, and parents, as well as practitioners, could benefit from knowing the pros and cons of experiences of immersion in a virtual environment.

## 5. Conclusions and Study Limitations

To the best of our knowledge, this is the first study that ‘maps’ a transdisciplinary field of inquiry to identify gaps and suggest future directions for research. We show that research on XR and children has substantially increased since 2017; the availability and affordability of hardware and software for immersive experiences have contributed to this trend. With most of the studies focused on children in their middle childhood (8–12 years of age), with an overrepresentation of empirical research studies on interventions, treatments, and medical procedures in clinical contexts using VR, this comprehensive literature review can provide a useful contribution to re-orient future research with children and XR. It can also provide clinical practitioners working with children with a snapshot on the state of the art in XR research and access to useful applications of these emerging technologies in clinical contexts.

This literature review has some limitations. Firstly, due to the number of resources and the transdisciplinary nature of the contribution, we did not provide an in-depth qualitative analysis of each strand of studies. Since the aim of this paper was to provide a snapshot of research trends on XR and children across different disciplines, we suggest that readers consider every category or strand as a small-scale literature review in a specific area concerned with XR and children. In fact, we encourage researchers to build upon the four strands and to dive deeper into the different areas we highlighted. We trust every strand will continue to grow in the future, perhaps at a different pace to that shown by our figures.

Secondly, given the large number of sources that were identified and the time it took to engage in the analysis of this work, as well as the incredible pace at which innovation occurs in the field of XR research and development, this review is already out of date by default at the time it is published. Therefore, we advise researchers to take our conclusions not as definitive statements, but rather as ‘orientations’ that can contribute to making more informed decisions when approaching such a complex field. For instance, researchers could screen the literature starting from 2021 and focus on one of the four research stands; meanwhile, clinical practitioners, interested in innovating their practice using XR technologies, can find important resources on how to reduce pain perception using HMD. More specifically, the four strands we have identified and the references reported in this review can orient pediatricians and other healthcare professionals to identify authors who have written and worked on specific XR procedures and check whether those authors have carried out more recent work and contact them directly. In order words, the reorganization of this substantial material on XR and children can allow readers to access information more easily a more quickly and build upon the strands to start or continue their investigations in this field.

Moreover, since many insights in this field constitute intellectual property protected as trade secrets, it is reasonable to assume that a large amount of knowledge that is available in principle cannot be easily or systematically assessed. The consultation of the ‘grey literature’, such as research reports, can be an option for overcoming this limitation. Some tech companies allow access to their research findings, especially when partnering with public research institutions. However, this puts into perspective the challenges associated with the fast-paced, industrial development of technology for commercial exploitation, and the potential societal effects experienced by users, in this case children, and raises the following question: how can we best protect and support the interests of users? Hopefully, we will be able to see more research on children’s perspective on the Metaverse and their experiences in immersive environments not only in terms of comfort/discomfort, satisfaction/dissatisfaction, or any other physiological indicators, but also work that treats children as co-creators of technologies that can better respond to their rights to learning, entertainment, healthcare, etc. If the Cyborgs are already among us, or we all already are Cyborgs in some ways, it will be important to grow savvier to better understand how to educate our children to best interact and possibly harness the potential of technologies that, already, have become integrated and embodied in our lives.

## Figures and Tables

**Table 1:** Search terms and results of the literature search.

Search Terms	Results
“AR” + child-	5170
“Virtual Reality” + child-	3493
“VR” + child-	1400
“Augmented reality” + child-	1210
immersive + child-	836
**TOTAL**	**12,109**

**Table 2:** Research strands on children’s experiences in XR (*n* = 485).

Type of Technology	Clinical Interventions	Teaching and Learning	Adoption and User Experience	Design and Prototyping
HMD	131	55	19	12
HMD vs. other tech or treatments	40	21	2	1
CAVE	29	15	10	4
Screen-based VR	43	38	9	9
AR	8	15	2	9
Virtual environment (projections)	0	9	2	2
**Total**	**251**	**153**	**44**	**37**

**Table 3:** Correspondence between QCA and Leximancer analysis.

Themes Emerging from Content Analysis	Leximancer Concepts
Clinical Interventions	Intervention, patients, risk
Teaching and Learning	Crossing, learning, parents, social
Adoption and User Experience	Body, children, environment
Design and Prototyping	Design, VR

## Data Availability

The original contributions presented in the study are included in the Supplementary Materials, further inquiries can be directed to the corresponding author.

## Supplementary Materials

The following supporting information can be downloaded at: https://www.mdpi.com/article/10.3390/children11080984/s1, Table.
